# The epigenetic clock and pubertal, neuroendocrine, psychiatric, and cognitive outcomes in adolescents

**DOI:** 10.1186/s13148-018-0528-6

**Published:** 2018-07-18

**Authors:** Anna Suarez, Jari Lahti, Darina Czamara, Marius Lahti-Pulkkinen, Polina Girchenko, Sture Andersson, Timo E. Strandberg, Rebecca M. Reynolds, Eero Kajantie, Elisabeth B. Binder, Katri Raikkonen

**Affiliations:** 1 Department of Psychology and Logopedics, University of Helsinki, Haartmaninkatu 3, PO Box 21, FI-00014 Helsinki, Finland; 2 Helsinki Collegium of Advanced Studies, University of Helsinki, 00014 Helsinki, Finland; 3 Department of Translational Research in Psychiatry, Department of Psychiatry and Behavioral Sciences, Max-Planck Institute of Psychiatry, 80804 Munich, Germany; 4 Department of Psychiatry and Behavioral Sciences, School of Medicine, Emory University, Atlanta, 30322 USA; 5 National Institute for Health and Welfare, Helsinki and Oulu, 00271 Helsinki, Finland; 6 Children’s Hospital, Helsinki University Central Hospital and University of Helsinki, 00029 Helsinki, Finland; 7 Center for Life Course Health Research, University of Helsinki, Geriatrics, Helsinki University Hospital, University of Oulu, 00029 Helsinki, Finland; 8 BHF Centre for Cardiovascular Science, Queen’s Medical Research Institute, University of Edinburgh, Edinburgh, EH16 4TJ UK

## Abstract

**Background:**

Molecular aging biomarkers, such as epigenetic age predictors, predict risk factors of premature aging, and morbidity/mortality more accurately than chronological age in middle-aged and elderly populations. Yet, it remains elusive if such biomarkers are associated with aging-related outcomes earlier in life when individuals begin to diverge in aging trajectories. We tested if the Horvath epigenetic age predictor is associated with pubertal, neuroendocrine, psychiatric, and cognitive aging-related outcomes in a sample of 239 adolescents, 11.0–13.2 years-old.

**Results:**

Each year increase in epigenetic age acceleration (AA) was associated with 0.06 SD units higher weight-for-age, 0.08 SD units taller height-for-age, -0.09 SD units less missed from the expected adult height, 13 and 16% higher odds, respectively, for each stage increase in breast/genitals development on the Tanner Staging Questionnaire and pubertal stage on the Pubertal Development Scale, 4.2% higher salivary cortisol upon awakening, and 18 to 34% higher odds for internalizing and thought problems on the Child Behavior Checklist (*p* values <  0.045). AA was not significantly associated with cognition.

**Conclusions:**

Our findings suggest that already in adolescence, AA is associated with physiological age acceleration, which may index risk of earlier aging. AA may identify individuals for preventive interventions decades before aging-related diseases become manifest.

**Electronic supplementary material:**

The online version of this article (10.1186/s13148-018-0528-6) contains supplementary material, which is available to authorized users.

## Background

There is an imperative to discover biomarkers of human aging, driven by demographic shifts and the striking inter-individual variation in trajectories in aging [1, 2]. Biomarkers of aging would allow more precise quantification of true biological age, permitting the identification of individuals at risk of aging-related diseases for preventive interventions decades before disorders become manifest.

Among the most promising molecular biomarkers of aging are those based on changes in DNA methylation (DNAm), the covalent addition of a methyl group primarily to cytosine linked to guanine by phosphate (CpG) sites [3]. Variation in DNAm of 71 CpG sites in the whole blood [4] and of 353 CpG sites from multiple tissues and cell types [5] have been identified to predict chronological age with high accuracy (*r* > .91); note that the algorithms involve both increased and decreased methylation of CpG sites to predict aging. These, Hannum and Horvath epigenetic age predictors, demonstrate a median absolute difference between DNAm age and actual chronological age of up to 4.9 [4] and 3.5 years [5] and have been validated in 19–101 [4] and 0–100-year-old [5] individuals, respectively. In middle-aged and elderly individuals, the Horvath and Hannum-based measures of epigenetic age acceleration (AA) (higher DNAm age than actual chronological age) were associated with higher body-mass index [6], lower physical and cognitive fitness [7], Alzheimer’s disease [8], menopause [9], and increased risk of all-cause mortality [10, 11].

Studies in middle-aged to elderly populations are, however, confounded by the often decade-long processes of aging-related disease and aging in itself. Therefore, studies of aging might better focus earlier in life, when inter-individual differences in aging trajectories start to emerge, but before most age-related diseases become manifest [12]. Such studies focusing on AA early in life are scarce. In one study, which tested associations between the Horvath epigenetic age predictor at birth, 7 and 17 years and physical growth and development among 400 to 1000 UK children, found that higher AA at birth predicted higher fat mass in childhood and adolescence, faster growth in weight and body mass index (BMI), slower growth in fat mass, and higher odds of increasing Tanner stage of testes development between childhood and adolescence [13]. The same study also found that AA at age 7 was associated with increased height in childhood and adolescence, but slower growth in height between childhood and adolescence [13], suggesting earlier physiological maturation. In a study of 46 US adolescent girls using the Horvath epigenetic age predictor, AA at age 13 years was associated with higher salivary cortisol [14].

There are few data on associations between epigenetic age and aging-related biomarkers early in life. There is an absence of literature of other early life phenotypes well-known to be related to aging-related diseases and/or premature mortality, namely, psychiatric problems and cognitive functioning [15, 16]. We examined 11.0–13.2-year-old Finnish adolescents to determine whether the Horvath epigenetic age predictor is related to the tempo of markers of physical growth and development, hypothalamic-pituitary-adrenal (HPA) axis functioning, psychiatric problems, and cognition. Based on preclinical studies showing that fetal exposure to excess glucocorticoids ‘programs’ an offspring phenotype of accelerated risk factors for cardiometabolic and psychiatric disorders, likely mediated, in part, via altered DNAm profiles [17, 18], we hypothesized that AA would be associated with more advanced physical growth and development, higher diurnal and lesser suppressed salivary cortisol in response to dexamethasone, higher total and internalizing and externalizing psychiatric problems, and lower scores on neuropsychological tests of intelligence.

## Results

**Fig. 1:**
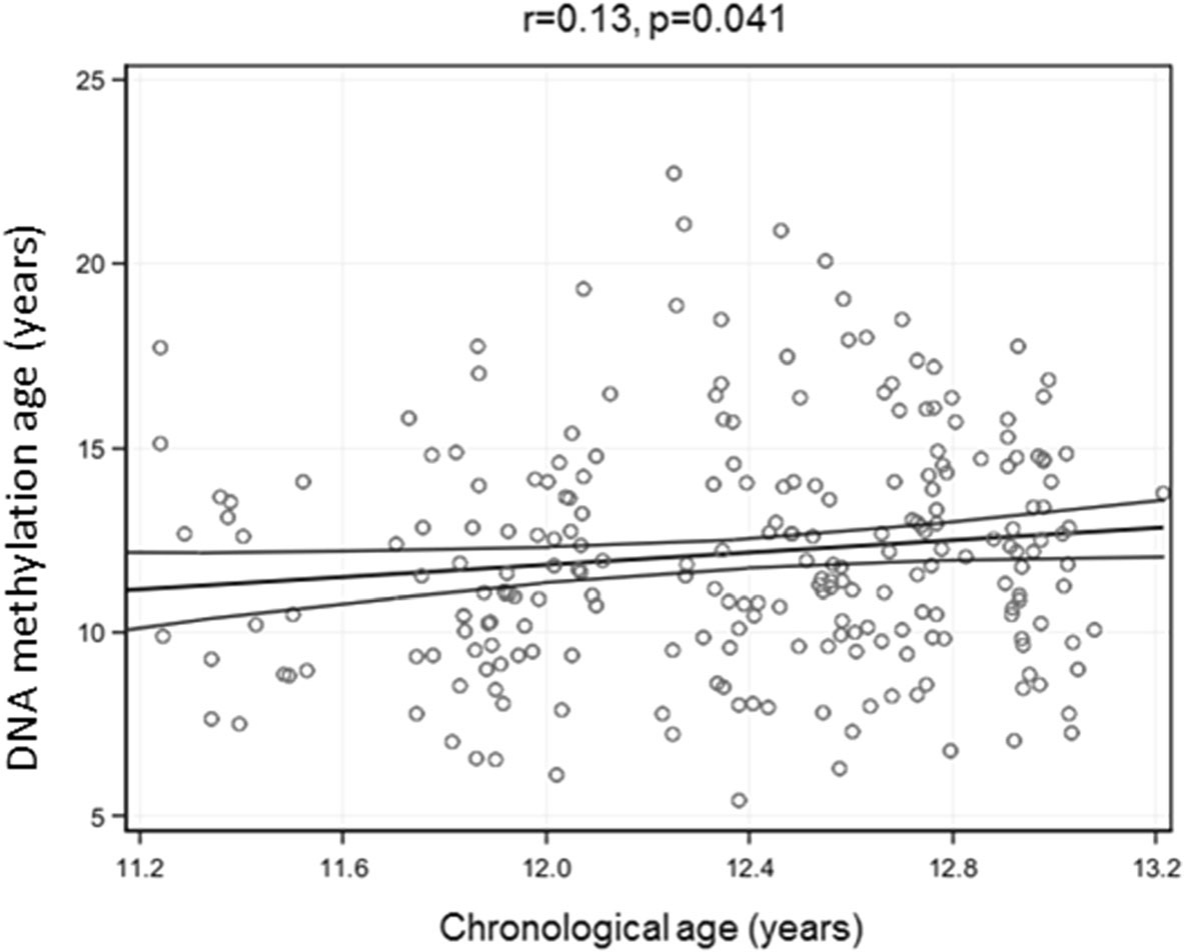
A scatterplot with a regression line and 95% confidence intervals showing associations between DNA methylation age and chronological age in 11.0–13.2-year-old adolescents

Characteristics of the sample are in Table 1. Pearson correlations between DNAm age and chronological age was 0.13 (*p* = 0.041) (Fig. 1). Pubertal (Pearson’s *r* |0.28–0.91|), neuroendocrine (|0.41–0.58| *p* < 0.001; salivary cortisol awakening response and nadir *r* = − 0.02,*p* = 0.74), psychiatric (0.14–0.71 *p* < 0.05; DSM-IV-oriented somatic and oppositional defiant problems *r* = 0.13,*p* = 0.06), and cognitive (0.48–0.85 *p* < 0.001) outcomes were correlated. Of the covariates, none was significantly associated with the adolescent AA (Additional file 1: Table S1).

**Table 1:** Characteristics of the sample

Child characteristics at birth:	*N*	Mean/*N*	SD/%	Range
Sex (boys)	239	116	48.5	
Length of gestation (weeks)	239	40.0	1.3	35.6–42.4
Birth weight (g)	239	3571	472	1890–4995
Birth length (cm)	239	50.3	1.9	43.0–55.0
Birth order	239			
First		136	56.9	
Second or later		103	43.1	
Child characteristics in adolescence:				
Chronological age (years)	239	12.4	0.5	11.1–13.2
DNA methylation age (years)	239	12.1	3.1	5.4–22.5
Weight (kg)	238	48.7	10.8	23.2–91.1
Weight-for-age (SD)	238	0.3	1.0	− 3.6 – 3.0
Height (cm)	238	156.2	7.5	134.5–177.0
Height-for-age (SD)	238	0.2	1.0	− 2.7 – 2.5
Body mass index (kg/m^2^)	238	19.8	3.44	12.8–34.5
Body-mass-index-for-age (SD)	238	0.3	1.0	− 3.1 – 2.7
Target height (SD)	238	173.4	7.6	153.9–191.7
Mid-parental target height (SD) minus height-for-age (SD)	238	0.5	1.0	− 1.9 – 3.5
Tanner Staging Questionnaire				
Pubic hair development	233			
I		50	21.5	
II		83	35.6	
III		71	30.5	
IV		29	12.4	
Breast/genitalia development	234			
I		18	7.7	
II		81	34.6	
III		83	35.5	
IV		52	22.2	
Pubertal development scale	236			
No development		139	58.9	
Development barely begun		74	31.4	
Development definitely under way		23	9.7	
Diurnal salivary cortisol				
ln(upon awakening)	218	1.7	0.6	− 1.7 – 3.6
ln(awakening response)	218	0.4	0.7	− 0.9 – 6.3
ln(Nadir)	218	− 0.9	0.9	− 2.3 – 1.8
ln(response to dexamethasone suppression test)	213	0.4	0.8	−v5.2 – 3.0
Psychiatric problems (> 82nd percentile borderline clinically significant problems)	222			
Total behavior problems		38	17.1	
Internalizing problems		18	8.1	
Externalizing problems		25	11.3	
Intelligence quotient, estimated (*M* = 100; SD = 15)*				
General	235	105.8	14.8	57–140
Verbal	236	111.4	17.2	57–153
Performance	237	100.9	19.7	42–141
Maternal characteristics:				
Age at delivery (years)	239	30.4	4.4	20.0–43.0
Weight at delivery (kg)	237	63.4	9.9	45.0–98.0
Height (cm)	239	166.8	5.5	151.0–180.0
Body mass index at delivery (kg/m^2^)	237	22.8	3.5	16.5–36.0
Mode of delivery	239			
Vaginal		214	89.5	
Cesarean		25	10.5	
Alcohol consumption during pregnancy	239			
No		199	83.3	
Yes		40	16.7	
Smoking during pregnancy	239			
No		217	90.8	
Yes		22	9.2	
Consumption of glycyrrhizin in licorice during pregnancy	239			
Low (0–249 mg/week)		174	72.8	
Medium (250–499 mg/week)		33	13.8	
High (≥ 500 mg/week)		32	13.4	
Age at menarche (years)	228			
Mothers of girls	120	12.69	1.39	9.0–16.0
Mothers of boys	108	12.70	1.19	10.0–16.0
Paternal/parental characteristics:				
Paternal height (cm)	236	180.07	6.38	157.00–200.00
Highest educational level of either parent at child’s adolescence follow-up	239			
Secondary or less		25	10.5	
Vocational		59	24.7	
University degree		155	64.9	

*Note*: *3, 3, and 7 children had estimated general, verbal, and performance intelligence quotient below 70 because of difficulties in visual processing

### AA and physical growth and development

Table 2 shows that in models adjusted for adolescent’s sex and the first three MDS components (model 1), each year increase in AA was associated with 0.06 SD unit higher weight-for-age, 0.08 SD unit taller height-for-age, and − 0.09 SD units less missed from the target adult height (*p* values < 0.02). Also each year increase in AA was associated with a more advanced Tanner stage of breast/genitals development and a more advanced pubertal stage on the PDS (13 and 16% odds to increase in stage per each year increase in AA, respectively; *p* values < 0.018). When adjusted for the other covariates (model 2) the associations remained significant (*p* values < 0.014) except for weight-for-age SD score, which became non-significant (*p* = 0.051) (Table 2). When we made adjustments for maternal self-reported age at menarche, all the significant associations remained significant (*p* < 0.042; data not shown). When corrected for multiple testing (tests across 7 outcomes), the association with height-for-age SD score (Bonferroni-corrected *p* = 0.035) and own current height-for-age SD score minus mid-parental target height SD score (Bonferroni-corrected *p* = 0.007) remained significant.

**Table 2:** Associations between epigenetic age acceleration and growth and physical development and in 11.0–13.2-year-old adolescents

Outcome:	Epigenetic age acceleration (years) (unstandardized residual regressing DNA methylation age on chronological age and blood cell count types)					
	Model 1			Model 2		
	B/OR	95% CI	*p*	B/OR	95% CI	*p*
Anthropometry						
Weight-for-age (SD)	0.06	0.01; 0.11	0.02	0.05	0.00; 0.10	0.051
Height-for-age (SD)	0.08	0.03; 0.13	0.003	0.07	0.02; 0.12	0.01
Body-mass-index-for-age (SD)	− 0.04	− 0.01; 0.09	0.15	0.02	− 0.02; 0.07	0.31
Mid-parental target height (SD) minus height-for-age (SD)	− 0.09	− 0.14; − 0.04	0.001	− 0.09	− 0.14; − 0.03	0.001
Tanner Staging Questionnaire						
Pubic hair development (I–IV)	1.09	0.98; 1.21	0.12	1.15	0.99; 1.25	0.07
Breast/genitals development (I–IV)	1.13	1.02; 1.25	0.018	1.15	1.03; 1.29	0.014
Pubertal development scale						
Stage (I–III)	1.16	1.02; 1.32	0.015	1.19	1.05; 1.34	0.008

*Note*: B refers to unstandardized regression coefficient from generalized model with Gaussian reference distribution; OR refers to odds ratio from generalized linear model with ordinal logistic reference distribution; 95% CI refers to 95% confidence interval

Model 1 is adjusted for adolescent sex and the first three multidimensional scaling components based on genome-wide data; model 2 is adjusted for model 1 covariates plus birth weight, gestational age, parity, delivery mode, maternal age and body mass index at delivery, maternal smoking, alcohol and glycyrrhizin in licorice use during pregnancy, and highest achieved education of either parent in adolescence follow-up

None of the physical growth associations varied by sex (*p* values > 0.052 for sex × AA interactions; data not shown).

### AA and diurnal and dexamethasone suppressed salivary cortisol

**Fig. 2:**
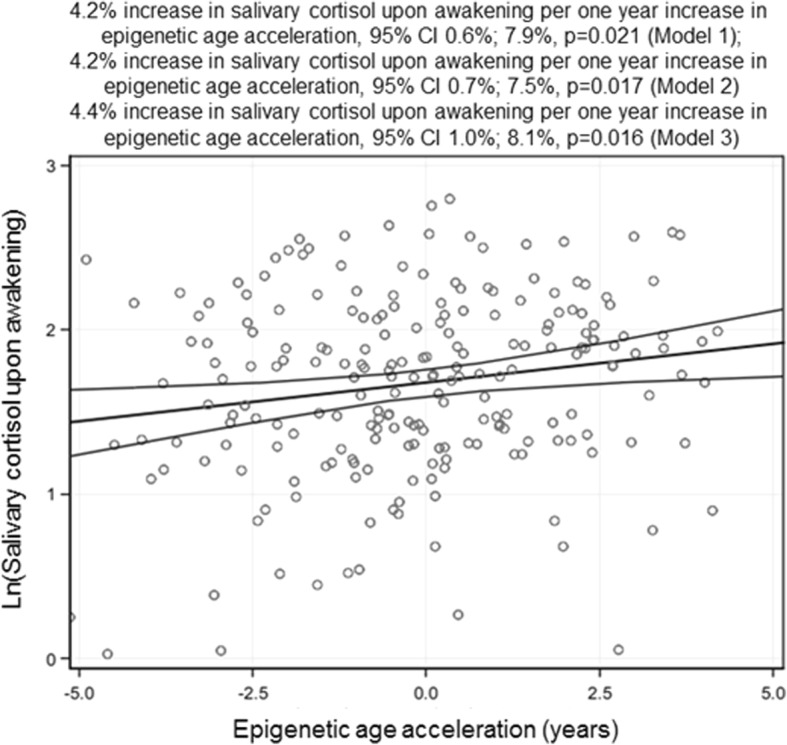
A scatterplot with a regression line and 95% confidence intervals showing associations between epigenetic age acceleration and salivary cortisol upon awakening in 11.0–13.2-year-old adolescents. Epigenetic age acceleration is calculated as the residual from a linear regression where DNA methylation age is regressed on chronological age and adjusted for six cell types. Numbers showing percent increase in salivary cortisol upon awakening per 1 year increase in epigenetic age acceleration and 95% confidence intervals are derived from generalized linear models with Gaussian reference distribution and adjusted for three multidimensional scaling components from genome-wide data, adolescent’s sex, and time upon awakening (model 1); and model 1 plus birth weight, gestational age, parity, delivery mode, maternal age and body mass index at delivery, maternal smoking, alcohol and glycyrrhizin in licorice use during pregnancy, and highest achieved education of either parent in adolescence follow-up (model 2); and model 2 plus body-mass-index-for-age SD score in adolescence (model 3)

In models adjusting for adolescent’s sex, the first three MDS components and time at awakening, for each year increase in AA, salivary cortisol at awakening increased by 4.2% (95% CI 0.6; 7.9, *p* = 0.021) (Fig. 2). This association survived covariate adjustments (model 2), and adjustments for the adolescents BMI-for-age SD score (Fig. 2; *p* values < 0.02), but not correction for multiple testing (tests across 4 cortisol outcomes; Bonferroni-corrected *p* = 0.08). Higher AA also associated with salivary cortisol awakening response, but this association was sex-specific (*p* = 0.02 for sex × AA interaction). Additional file 2: Figure S1 shows that in boys, for each year increase in AA, salivary cortisol awakening response decreased by − 4.9% (95% CI − 10.4; 0.09, *p* = 0.06), while in girls it increased by 3.1% (95% CI − 1.0; 7.3, *p* = 0.11); in neither of the groups was the change in salivary cortisol awakening response significant. There were no other significant associations with the salivary cortisol parameters (*p* values > 0.06) and no other sex interactions (*p* values> 0.06) (data not shown).

### AA and psychiatric problems

**Fig. 3:**
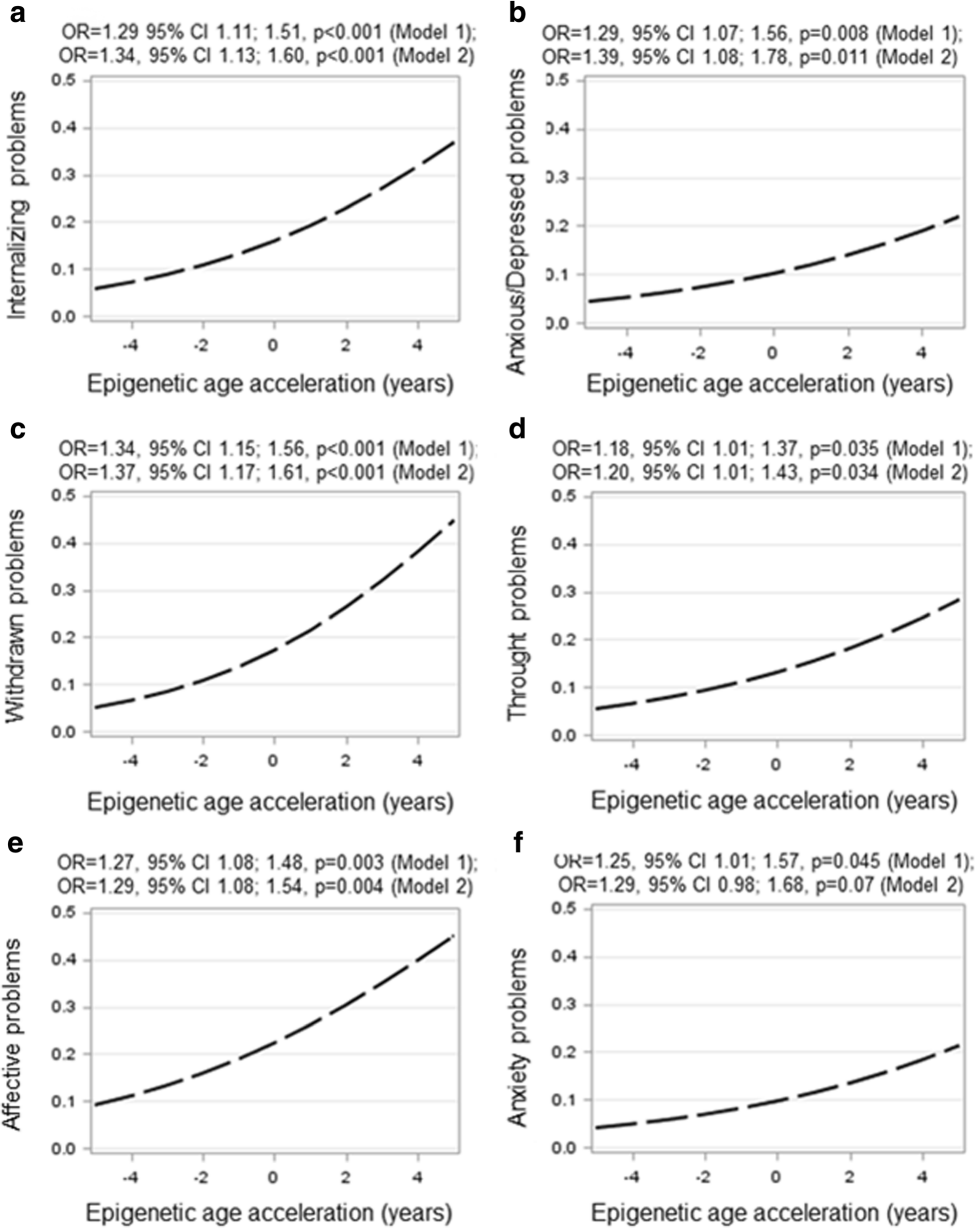
Predicted probability of having borderline clinically significant psychiatric problems (panel **a**: internalizing problems, panel **b**: anxius/depressed problems, panel **c**: withdrawn problems, panel **d**: thought problems, panel **e**: affective problems, panel **f**: anxiety problems) according to epigenetic age acceleration in 11.0–13.2-year-old adolescents. Epigenetic age acceleration is calculated as the residual from a linear regression where DNA methylation age is regressed on chronological age and adjusted for 6 cell types. Odds Ratios (OR) and 95% Confidence Intervals are derived from generalized linear models with binary logistic reference distribution and adjusted for three multidimensional scaling components from genome-wide data, and adolescent’s sex (model 1); and model 1 plus birth weight, gestational age, parity, delivery mode, maternal age and body mass index at delivery, maternal smoking, alcohol and glycyrrhizin in licorice use during pregnancy and highest achieved education of either parent in adolescence follow-up (model 2)

Figure 3 shows that after the model 1 covariate adjustments, each year increase in AA was associated with 29% higher odds for internalizing problems, and 29 and 34% higher odds for anxious/depressed and withdrawn problems on the internalizing problems domain, respectively (*p* values< 0.008); each year, increase in AA was associated with 27 and 25% higher odds for DSM-IV oriented affective and anxiety problems (*p* values< 0.045); and each year, increase in AA was also associated with 18% higher odds for thought problems within the domain of other problems (*p* = 0.035). When adjusted further for the other covariates (model 2), the association of AA with anxiety problems became non-significant (*p* = 0.07), while the other significant associations remained unaffected (*p* values < 0.034) (Fig. 3). When corrected for multiple testing (tests across 4 internalizing, 6 DSM-IV, and 3 other problems domains), the associations with internalizing, anxious/depressed, withdrawn, and affective problems (Bonferroni-corrected *p* value< 0.018) remained significant.

There were no other significant associations with child psychiatric problems (*p* values> 0.09; data not shown), and no significant sex differences in these associations (*p* values> 0.06 for sex × AA interactions; data not shown).

### AA and cognition

There were no significant associations between AA and estimated intelligence (Additional file 1: Table S2).

## Discussion

As we hypothesized, adolescents with higher AA, meaning higher DNAm age than chronological age, was associated with more advanced physical growth and development, higher salivary cortisol, and higher odds for psychiatric problems. Adolescents with higher AA were heavier- and taller-for-age, and closer to their expected target adult height, suggesting an earlier growth spurt and less remaining growth potential. Their pubertal stage of breast/genital development, according to the Tanner Staging Questionnaire and of secondary sex characteristics according to PDS, were also at a more advanced stage.

The advanced growth and maturation was accompanied by higher salivary cortisol upon awakening, and they had higher odds for displaying borderline clinically significant internalizing problems, in particular anxious/depressed and withdrawn problems, and affective and anxiety as well as thought problems. While these associations remained significant after adjustments for a number of important covariates, including maternal pregnancy and child perinatal characteristics and genetic population structure, only the associations with stature and with internalizing/affective problems remained significant after correction for multiple testing. Yet, the Bonferroni-correction may be an overly stringent method to account for the multiple testing problem here, as the outcomes, within each developmental domain were not independent of each other. These results thus suggest that adolescents whose biological DNAm age is more advanced than their chronological age display physiological age acceleration that may indicate risk of earlier aging.

Our findings are consistent with the life history theory that suggests that early development and early puberty are meaningful tradeoffs in conditions of environmental adversity [19, 20]. We suggest that AA and advanced growth and maturation are indicators of more advanced tempo of aging processes present from early life onwards. Indeed, more advanced physical growth and pubertal development have been shown to predict aging-related diseases, including cancers [21], cardio-metabolic disorders and their risk factors [22], and depression [23]. While associations between cortisol, the effector hormone of the HPA axis, and health are complex, in general high cortisol concentrations, are associated with a number of physical and mental health adversities, such as obesity [24], sleep problems [25], anxiety, and depression [26] in studies in both children and adults. Moreover, longitudinal studies suggest that high cortisol levels in late adulthood increase the risk for cardiometabolic disorders and cardiovascular mortality [27, 28]. While childhood psychiatric problems are associated with a number of physical health adversities and psychosocial problems [15], there is evidence that they tend to track into adulthood [29]. Even in those children whom psychiatric problems do not persist into adulthood or are subthreshold [30], and in those whom childhood psychiatric problems are parent-rated [31], are at increased risk for adverse adulthood outcomes, and hence, are likely more vulnerable for earlier aging.

Our findings with physical growth agree with one previous study [13] which demonstrated that higher AA at age 7 was associated with increased height in childhood and adolescence. Yet, the study found that higher AA at age 7 predicted slower, rather than faster height growth between childhood and adolescence, and that AA at ages 7 and 17 years was not associated with a number of markers of pubertal development, including peak height velocity and the Tanner Staging Questionnaire [13]. The previous study also found that higher AA at birth predicted higher fat mass, faster growth in weight and BMI, slower growth in fat mass, and higher odds of increasing Tanner stage of testes development between childhood and adolescence [13]. As DNAm undergoes age-related changes [32], a potential explanation for the somewhat discrepant study findings on physical growth and development between our and the previous study is the age-stage at which DNAm and physical growth were measured. The difference in findings does not, however, relate to tissue type as both studies measured DNAm in childhood/adolescence from venous blood. Yet, the *p* values in the previous study were at their best around 0.007, and none of the associations would have survived Bonferroni-correction for multiple testing.

Our findings for salivary cortisol are also in partial agreement with one previous study in adolescent girls, which demonstrated that higher AA, measured from salivary DNA, was associated with higher salivary cortisol measured across 2 days. This study did not, however, account for covariates, except for adolescent’s age at testing, and neither of the previous studies accounted for genetic population structure, which is strongly associated with methylation profiles [33]. Hence, it remains unclear if the findings of the latter would have survived adjustments for important covariates, such as BMI, and if both of these previous studies would have survived adjustments for genetic population structure.

Our study also revealed novel findings related to psychiatric problems, namely, that higher AA was associated with internalizing/affective-type and thought problems, but not externalizing-type of problems. This finding may reflect statistical power, as internalizing problems in our sample were twice as prevalent as externalizing problems, or studying adolescents, a time when a marked rise in internalizing problems is observed [34]. This pattern is, however, congruent with our other recent study. Interestingly, we have demonstrated that another epigenetic age biomarker, namely, lower DNAm gestational age than chronological gestational at birth predicts higher internalizing, but not externalizing problems in early childhood, though in a sex-specific manner such that boys are more vulnerable [35]. AA was not associated with intelligence. This is somewhat surprising, as poorer childhood cognitive functioning is predictive of aging-related diseases, including dementia [16]. Hence, because of the developmental changes in DNAm [32], we cannot rule out that AA at later developmental stages will change and become associated with cognitive function and perhaps change its links to behavioral problems.

### Strengths

The strengths of our study relate to a well-characterized cohort and availability of a number of aging-related phenotypes that we measured decades before the aging-related diseases become manifest. We were also able to account for a number of early life adversities and their proxies, such as maternal smoking and alcohol use during pregnancy, maternal age and BMI at delivery, mode of delivery, and the adolescent’s birth weight and gestational age. We also accounted for maternal glycyrrhizin in licorice use during pregnancy, which is a potent inhibitor of the placental glucocorticoid barrier enzyme (11-beta hydroxysteroid dehydrogenase type 2) and which may result in fetal overexposure to maternal circulating glucocorticoids; our study was originally designed to examine its associations with offspring developmental outcomes, and it is commonly consumed in young Finnish women (in our original cohort nearly 50%) [36]. None of these early life factors were significantly associated with epigenetic AA in this sample, which is contrary to what we expected based on previous findings by us and others showing that these factors may exert adverse consequences on offspring neurodevelopment and HPA-axis functioning [37, 38]. With regard to maternal glycyrrhizin use, we have previously shown in a larger sample of this study cohort that high maternal intake of glycyrrhizin in licorice during pregnancy (> 500 mg/week) is associated with slightly shorter length of gestation [39], poorer performance in neurocognitive tests at ages 8 and 11–13 [40, 41], higher odds for having borderline clinically significant externalizing psychiatric problems at ages 8 and 11–13 [40, 41], higher diurnal and stress-induced salivary cortisol profiles at age 8 [42], and more advanced pubertal maturation in girls at age 11–13 [41]. Whether maternal intake of glycyrrhizin in licorice during pregnancy is associated with other epigenetic biomarkers than the epigenetic age biomarker studied here, awaits further investigation.

### Limitations

The limitations of our study are the narrow age range of our sample, and hence, the small magnitude of the correlation between DNAm age and chronological age. The small magnitude of this correlation is, however, similar to the other two previous childhood epigenetic age studies [13, 14]. The narrow age range also limits generalizability from our findings to samples that differ in age from ours as DNAm changes with age. Our findings are also limited to DNAm in one tissue type and our study precludes generalizations beyond Finnish children. Further, our study design was cross-sectional which precludes testing developmental changes and causal inferences. We can neither address the possibility of selection bias resulting from sample attrition to genetic analyses. Also measuring genome-wide methylation with the most recent Illumina EPIC array that lacks 16 (4.5%) of the 353 CpG sites originally needed for Horvath DNAm age calculation which should be kept in mind when interpreting the study findings. Finally, while we measured psychiatric problems with a standardized, validated, and widely used mother-report [43], we cannot rule out potential information-bias embedded in the mother-report. Hence, future studies need to confirm whether our study findings on psychiatric problems also pertain to clinical diagnoses.

## Conclusions

Our study shows that among 11.0–13.2-year-old adolescents, AA is associated with a number of markers that index risk for earlier aging, namely, more advanced physical growth and development, higher salivary cortisol upon awakening, and psychiatric problems. Our findings are consistent with the life history theory [19, 20] and lend credence to the proposition that AA may be used as a biomarker of aging already early in life [3, 13].

## Methods

### Study population

*Glycyrrhizin in Licorice (GLAKU)* is an urban community-based cohort comprising originally 1049 women and their healthy, singleton infants born in 1998 in Helsinki, Finland [39]. Between 2009 and 2011, all initial cohort members who had given permission to be contacted, and whose addresses were traceable (*N* = 920, 87.70% of the original cohort in 1998) were invited to a follow-up. Of them, 692 (75.20%) could be contacted by phone, and 451 (65.20% of those contacted by phone) participated in a follow-up at the child’s mean age of 12.3 years (*SD* = 0.5, range 11.0–13.2 years). Of the participating adolescents, 243 donated blood for genetic analyses. After quality control procedures, 239 DNA samples remained for genetic analyses.

In comparison to those who participated in the follow-up but who did not donate blood or were excluded from genetic analyses based on quality control reasons (*n* = 212), the analytic sample with DNA (*n* = 239) had higher weight (mean difference (MD) = 3.27 kg, *p* < 0.01), height (MD = 2.72 cm, *p* < 0.01) and BMI (MD = 0.69 kg/m^2^, *p* = 0.03) at the adolescence follow-up, and their mothers had higher weight at delivery (MD = 1.76 kg, *p* = 0.05). The groups did not differ in chronological age at adolescent follow-up, sex, birth order, body size at birth, length of gestation, nor did their mothers differ in licorice or alcohol consumption or smoking during pregnancy, BMI at delivery or mode of delivery, age at menarche; their mothers and fathers did not differ in educational attainment or height (all *p* values> 0.07).

Ethics Committees of the City of Helsinki and the Uusimaa Hospital District approved the study protocol. Written informed consent was obtained from the mother at birth and from parent/guardian and adolescent at the follow-up.

### Adolescent DNA methylation, epigenetic age, and blood cell count composition

Blood samples were collected, and DNA was extracted according to standard procedures. Methylation analyses were performed at the Max Planck Institute of Psychiatry in Munich, Germany. DNA was bisulphite-converted using the EZ-96 DNA Methylation kit (Zymo Research, Irvine, CA). Genome-wide methylation status of over 850,000 CpG sites was measured using the Illumina Infinium MethylationEPIC arrays (Illumina Inc., San Diego, CA) according to the manufacturer’s protocol. The arrays were scanned using the iScan System (Illumina Inc., San Diego, CA). The final dataset contained 812,943 CpGs.

We obtained DNAm-predicted age based on Horvath method [5] using the online epigenetic clock calculator (http://labs.genetics.ucla.edu/horvath/dnamage/). This calculator also incorporates information on blood cell counts for 6 cell types (granulocytes, monocytes, natural killer cells, B cells, CD4+ T cells, and CD8+ T cells) based on the Houseman method [44]. Epigenetic age was calculated as the unstandardized residual from a linear regression of DNAm age on chronological age and 6 cell count types.

### Adolescent genotyping and multi-dimensional scaling analysis

Genotyping was performed on Illumina Human OmniExpress Exome 1.2 bead chip (Illumina Inc., San Diego, CA) at the Tartu University, Estonia in September 2014 according to the standard protocols. Genomic coverage was extended by imputation using the 1000 Genomes Phase I integrated variant set (v3/April 2012; NCBI build 37/hg19) as the reference sample and IMPUTE2 software. Before imputing the following QC, filters were applied: SNP clustering probability for each genotype > 95%, Call rate > 95% individuals and markers (99% for markers with MAF < 5%), MAF > 1%, HWE *p* > 1*10–6. Moreover, heterozygosity, sex check, and relatedness checks were performed and any discrepancies were removed (*N* = 2). We performed multi-dimensional scaling (MDS) analysis on the identity by state matrix of quality-controlled genotypes. The first three components depicted the origin admixture and were included as covariates in the regression analyses [33]. This information was available for 221 participants.

### Physical growth and development

We used the following three measures of growth and development: (i) the difference between the child’s height-for-age standard-deviation (SD) score based on Finnish growth charts [45] (using the current measured height without shoes, measured with a Seca stadiometer) (model 213; Seca GmbH & Co KG, Hamburg, Germany) and midparental target height in SD units [46]; this is a measure of remaining growth potential and, consequently, the timing of the pubertal growth spurt. (ii) The Tanner Staging Questionnaire administered by a research nurse. The questionnaire uses schematic drawings of two secondary sex characteristics (pubic hair development in girls and boys and breast development in girls and development of genitalia in boys) and yields two 5-stage scores ranging from pre-pubertal (stage I) to post-pubertal (stage V) [47]. (iii) The Pubertal Development Scale (PDS) is a self-report questionnaire on secondary sex characteristics (growth spurt, body hair that is not specifically pubic hair, and skin changes in girls and boys; menarche and breast development in girls; and facial hair and voice change in boys). The PDS yields one 4-stage score ranging from no development (I) to full completion of development (IV) [48].

We also measured weight in light clothing without shoes (model 8; Seca GmbH & Co KG) and calculated BMI (weight (kg)/height (m^2^)). We transformed the values into weight-for-age and BMI-for-age SD scores based on Finnish growth charts [45].

### Diurnal and dexamethasone-suppressed salivary cortisol

Saliva samples were collected using cotton swabs (Salivette; Sarstedt, Nümbrecht, Germany). The adolescents were asked not to eat, drink, or brush their teeth 30 min before each sample, with an exception of drinking a small amount of water. We also specifically asked them to restrain from any caffeine including drinks (coffee, tea, cola). On the first of two consecutive days, samples were collected upon awakening and 15, 30, 45, and 60 min thereafter, at 12:00 noon, at 5:00 p.m., and at bedtime. Dexamethasone was administered after the bedtime saliva sample, and a sample was collected upon awakening the next day. We used a low dose of dexamethasone (3 μg/kg of total body weight) to detect individual variation in hypothalamic-pituitary-adrenal (HPA) axis suppression [49]. Salivary cortisol concentrations were determined by solid-phase, time-resolved fluorescence immunoassay with fluorometric end-point detection (DELFIA; Wallac, Turku, Finland). The intraassay and interassay coefficients of variation varied between 4.0 and 7.7%, and the mean coefficient of variation between duplicate analyses was 5.9%.

Of the diurnal measures, we used/calculated the following parameters: cortisol at awakening, cortisol awakening response (peak value after awakening minus value upon awakening), nadir (minimum of diurnal values), and response to dexamethasone suppression test (value upon awakening on day two minus value upon awakening on day one).

### Psychiatric problems

Mothers completed the Child Behavior Checklist (CBCL/6-18), a standardized and validated rating scale screening for psychiatric problems [43]. The scale is hierarchically structured, such that it yields a total problems score, which is first subdivided into internalizing and externalizing problems scores, and then internalizing problems score is further subdivided into anxious/depressed, withdrawn, and somatic complaints scores, and externalizing problems into rule-breaking and aggressive behavior scores; scores not included in internalizing and externalizing problems score, but which are embedded in the total problems are social, thought, and attention problems [43]. The CBCL also yields six Diagnostic and Statistical Manual of Mental Disorders, Fourth Edition (DSM-IV)-oriented scores, namely, affective, anxiety, somatic, attention deficit hyperactivity, conduct, and oppositional-defiant problems scores [43]. Following the CBCL manual, we used the 82nd percentile as the cutoff to identify adolescents with borderline clinically significant problems [43].

### Cognitive function

We used the short form of the Wechsler-Intelligence-Scale-for-Children-III [50] which included vocabulary, similarities, block design, and picture arrangement subtests. We used age-standardized scores to estimate age-standardized total intelligence, and verbal and performance intelligence quotients (IQs) [51].

### Covariates and confounders

All analyses were adjusted for child’s sex and the first three MDS components to control for population structure (model 1). We made further adjustments for covariates previously associated with physical growth and development, salivary cortisol, psychiatric problems, and cognition in this cohort [41]: the highest educational level of either parent (secondary or less/vocational/university) reported at adolescent follow-up, maternal age (years), and BMI (kg/m^2^) at delivery calculated from weight and height derived from medical records, maternal smoking (no/yes), weekly alcohol (no/yes) and glycyrrhizin in licorice (0–249 mg/week, 250–499 mg/week, ≥ 500 mg/week) consumption during pregnancy, delivery mode (vaginal/cesarean), parity (primiparous/multiparous), gestational length (weeks) as confirmed by ultrasound scans, and birth weight (grams) of the adolescent, derived from birth records (model 2). In addition, we conducted analyses of pubertal maturation adjusting for maternal self-reported age at menarche (years) as a crude proxy of the genetic component of pubertal development. Analyses of HPA-axis activity were additionally adjusted for time at awakening and time at dexamethasone intake as well as for child’s BMI-for-age SD score.

### Statistical analyses

We use generalized linear models (GLM) to study associations between AA and outcomes, specifying Gaussian reference distribution for continuous (growth anthropometry, salivary cortisol, cognition), ordinal logistic for categorical (Tanner stages and PDS), and binary logistic reference distribution for dichotomous outcomes (psychiatric problems). All analyses were adjusted for covariates and confounders as described and we also tested if the associations between AA on outcomes varied by sex by including sex × AA interaction into the GLMs following main effects of these variables as the sexes may differ in epigenetic age, pubertal maturation, and the prevalence and etiology of psychiatric problems. We also report Bonferroni-corrected *p* values to account for multiple testing within each developmental domain. Statistical analyses were performed using IBM SPSS version 24.0.

## Additional files

Additional file 1: **Table S1.** Associations between epigenetic age acceleration and covariates in 11.0–13.2-year-old adolescents. **Table S2.** Associations between epigenetic age acceleration and cognition in 11.0–13.2-year-old adolescents. (DOCX 17 kb)

Additional file 2: **Figure S1.** A scatterplot with a regression lines showing associations between epigenetic age acceleration and salivary cortisol awakening response in 11.0–13.2-year-old adolescent boys and girls. Epigenetic age acceleration is calculated as the residual from a linear regression where DNA methylation age is regressed on chronological age and adjusted for 6 cell types. Numbers showing percent increase in salivary cortisol upon awakening per 1 year increase in epigenetic age acceleration and 95% confidence intervals are derived from generalized linear models with Gaussian reference distribution and adjusted for three multidimensional scaling components from genome-wide data and time upon awakening. (PPTX 38 kb)

## Abbreviations

95% CI: 95% confidence interval
AA: Epigenetic age acceleration
DNAm: DNA methylation
GLAKU: Glycyrrhizin in Licorice Study
OR: Odds ratio
SD: Standard deviation
