# Supplementary material for: The epigenetic clock and pubertal, neuroendocrine, psychiatric, and cognitive outcomes in adolescents
Source: Clin Epigenetics. 2018 Jul 18;10:96. doi: 10.1186/s13148-018-0528-6 (PMC6052515; doi:10.1186/s13148-018-0528-6)
Supplement: Supplementary file 1 — Table S1. Associations between epigenetic age acceleration and covariates in 11.0–13.2-year-old adolescents. Table S2. Associations between epigenetic age acceleration and cognition in 11.0–13.2-year-old adolescents. (DOCX 17 kb) [file 13148_2018_528_MOESM1_ESM.docx]

| **Table S1. Associations between epigenetic age acceleration and covariates in 11.0-13.2-year-old adolescents.** | | | |
| --- | --- | --- | --- |
| **Covariates** | **Outcome:**  **Epigenetic age acceleration (years)**  (unstandardized residual regressing DNA methylation age on chronological age and blood cell count types) | | |
| **Child characteristics at birth:** | **B** | **95% CI** | **p** |
| Sex (boy/girl) | -0.18 | -0.73; 0.37 | 0.51 |
| Gestational age (weeks) | -0.04 | -0.27; 0.15 | 0.71 |
| Birth weight (kg) | 0.30 | -0.34; 0.94 | 0.36 |
| **Parental characteristics:** |  |  |  |
| Maternal age at delivery (years) | 0.01 | -0.06; 0.07 | 0.88 |
| Maternal smoking during pregnancy (no vs. yes) | 0.44 | -0.50; 1.38 | 0.36 |
| Maternal alcohol consumption during pregnancy (no/yes) | 0.20 | -0.48; 0.89 | 0.56 |
| Maternal glycyrrhizin consumption in licorice during pregnancy (0-249/ vs. ≥250 mg/week) | 0.42 | -0.16;0.99 | 0.16 |
| Maternal body mass index at delivery (kg/m^2^) | -0.02 | -0.13; 0.08 | 0.65 |
| Parity (primiparous vs. multiparous) | -0.02 | -0.55; 0.59 | 0.94 |
| Delivery mode (vaginal vs. cesarean) | 0.60 | -0.52; 1.72 | 0.30 |
| Highest achieved education of either parent at adolescent follow-up (secondary or less vs. vocational/university) | 0.53 | -0.48; 1.54 | 0.31 |
| **Note**. B refers to unstandardized regression coefficient from generalized linear models with Gaussian reference distribution; 95% CI refers to 95% Confidence Interval. | | | |

| **Table S2. Associations between epigenetic age acceleration and cognition in 11.0-13.2-year-old adolescents.** | | | | | | |
| --- | --- | --- | --- | --- | --- | --- |
| **Outcome:** | **Epigenetic age acceleration (years)**  (unstandardized residual regressing DNA methylation age on chronological age and blood cell count types) | | | | | |
| **Wechsler Intelligence Scale for Children III** | **Model 1** | | | **Model 2** | | |
|  | **B** | **95% CI** | **p** | **B** | **95% CI** | **p** |
| General estimated intelligence | 0.003 | -0.02; 0.02 | 0.79 | 0.007 | -0.01; 0.03 | 0.50 |
| Verbal estimated intelligence | -0.003 | -0.02; 0.02 | 0.75 | 0.001 | -0.02; 0.02 | 0.94 |
| Performance estimated intelligence | 0.006 | -0.01; 0.02 | 0.40 | 0.009 | -0.01; 0.02 | 0.27 |
| **Note.** B refers to unstandardized regression coefficient from generalized linear models with Gaussian reference distribution; 95% CI refers to 95% Confidence Interval.  Model 1 is adjusted for adolescent sex and the first three multidimensional scaling components based on genome-wide data; Model 2 is adjusted for Model 1 covariates plus birth weight, gestational age, parity, delivery mode, maternal age and body mass index at delivery, maternal smoking, alcohol and glycyrrhizin in licorice use during pregnancy and highest achieved education of either parent in adolescence follow-up.  P-values>0.25 when 3, 3 and 7 children who had estimated general, verbal and performance intelligence quotient below 70 because of difficulties in visual processing, respectively. | | | | | | |
