# Supplementary figures and images for: The epigenetic clock and pubertal, neuroendocrine, psychiatric, and cognitive outcomes in adolescents
Source: Clin Epigenetics. 2018 Jul 18;10:96. doi: 10.1186/s13148-018-0528-6 (PMC6052515; doi:10.1186/s13148-018-0528-6)

## Slide 1
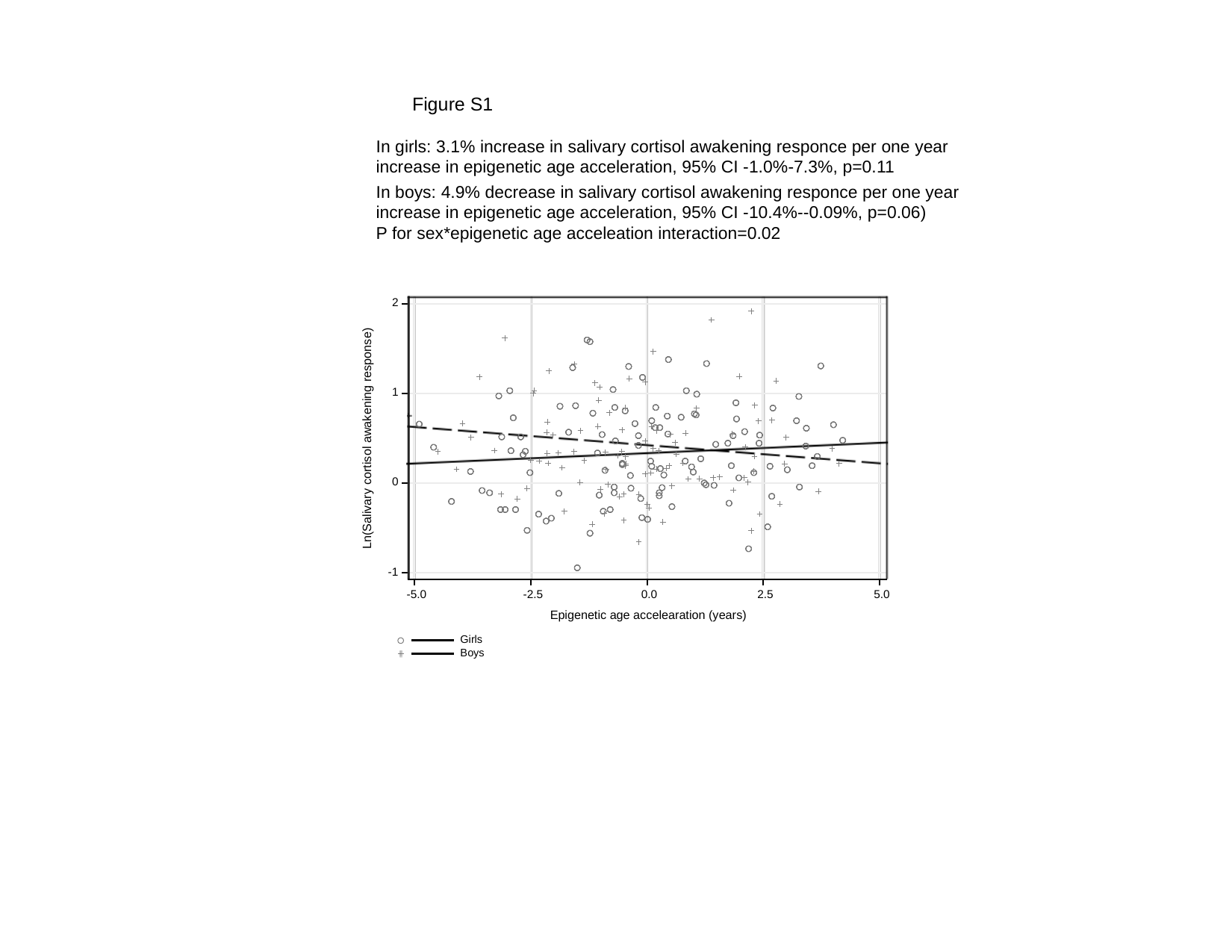

Supplement: Supplementary file 2 — Figure S1. A scatterplot with a regression lines showing associations between epigenetic age acceleration and salivary cortisol awakening response in 11.0–13.2-year-old adolescent boys and girls. Epigenetic age acceleration is calculated as the residual from a linear regression where DNA methylation age is regressed on chronological age and adjusted for 6 cell types. Numbers showing percent increase in salivary cortisol upon awakening per 1 year increase in epigenetic age acceleration and 95% confidence intervals are derived from generalized linear models with Gaussian reference distribution and adjusted for three multidimensional scaling components from genome-wide data and time upon awakening. (PPTX 38 kb) [file 13148_2018_528_MOESM2_ESM.pptx]
